# Widespread Myalgia and Chronic Fatigue: Phagocytes from Macrophagic Myofasciitis Patients Exposed to Aluminum Oxyhydroxide-Adjuvanted Vaccine Exhibit Specific Inflammatory, Autophagic, and Mitochondrial Responses

**DOI:** 10.3390/toxics12070491

**Published:** 2024-07-04

**Authors:** Jean-Daniel Masson, Ghidaa Badran, Romain K. Gherardi, François-Jérôme Authier, Guillemette Crépeaux

**Affiliations:** 1 Institut National de la Santé Et de la Recherche Médicale, Institut Mondor de Recherche Biomédicale, Université Paris Est Créteil, F-94010 Creteil, France; 2 Hôpitaux Universitaires Henri Mondor, Service d’Histologie/Centre Expert de Pathologie Neuromusculaire, Assistance Publique-Hôpitaux de Paris, F-94010 Creteil, France; 3 Ecole Nationale Vétérinaire d’Alfort, Institut Mondor de Recherche Biomédicale, F-94700 Maisons Alfort, France

**Keywords:** myalgic encephalomyelitis/chronic fatigue syndrome, macrophagic myofasciitis, aluminum-based adjuvant, autophagy/LC3-associated phagocytosis, inflammation, mitochondrial metabolism

## Abstract

(1) Background: Macrophagic myofasciitis (MMF) is an inflammatory histopathological lesion demonstrating long-term biopersistence of vaccine-derived aluminum adjuvants within muscular phagocytic cells. Affected patients suffer from widespread myalgia and severe fatigue consistent with myalgic encephalomyelitis/chronic fatigue syndrome (ME/CFS), a poorly understood disorder suspected to result from chronic immune stimulation by infectious and inorganic particles. (2) Methods: In this study we determined the immuno-metabolic properties of MMF phagocytic cells compared to controls, at rest and upon exposure to aluminum oxyhydroxide adjuvant, with or without adsorbed antigens, using protein quantification and an oxygen consumption assay. (3) Results: MMF and control cells similarly internalized the adjuvant and vaccine but MMF cells specifically expressed Rubicon and Nox2, two molecules unique to the LC3-associated phagocytosis (LAP) machinery, a non-canonical autophagic pathway able to downregulate canonical autophagy. MMF cells exhibited an altered inflammatory secretome, producing more pain-inducing CXC chemokines and less TNF-α than controls, consistent with chronic myalgia and exhaustion of the immune system previously documented in ME/CFS. MMF cells exhibited mitochondrial metabolism dysfunction, with exacerbated reaction to adjuvanted vaccine, contrasting with limited spare respiratory capacity and marked proton leak weakening energy production. (4) Conclusions: MMF phagocytes seemingly use LAP to handle aluminum oxyhydroxide vaccine particles, secrete pain-inducing molecules, and exhibit exacerbated metabolic reaction to the vaccine with limited capacity to respond to ongoing energetic requests.

## 1. Introduction

Myalgic encephalomyelitis/chronic fatigue syndrome (ME/CFS) is a poorly understood syndrome affecting millions of people worldwide, characterized by prolonged fatigue, malaise following exertion, foggy brain, and widespread arthromyalgia [1,2].

ME/CFS is multifactorial. It is reminiscent of post-infectious fatigue, e.g., fatigue following mononucleosis or long COVID [3,4], but could not be linked to a single infectious agent. Rather, it has been associated with a large panel of pathogens (viruses, intracellular bacteria causing Lyme disease, etc.) [2], but also with foreign intracellular particulate materials with immunostimulant properties (such as aluminum (Al) adjuvants of vaccines, silicone of leaky breast implants, tin of eroded sterilizing fallopian implants, etc.) [5,6,7]. Whether ME/CFS-triggering factors act by themselves or through reactivation of latent persistent viral infections [8] remains to be established [9]. Although a “hit and run” mechanism has been proposed, in which an acute infection resolves but leaves a dysfunctional immune system and/or autoimmunity [2], the classical hypothesis postulates that these changes are related to an inappropriate clearance of the immuno-stimulating triggers (e.g., living or dead pathogens, biopersistent foreign material). This may obviously cause “protracted immune stimulation that fails to switch off” [10], eventually leading to some “burn-out” of the immune system. This fits well with evidence that ME/CFS patients are flush with cytokines until around the three-year mark, at which point the immune system becomes exhausted and cytokine levels drop [11].

Clinical and laboratory features may slightly differ according to the type of trigger, and therefore, investigation of homogeneous subgroups of ME/CFS using validated experimental setups and well defined control groups has been recommended [2]. Integrated pathophysiological investigations of this immunologically driven multisystem disorder affecting mitochondrial metabolism, and neurologic and other functions are also required [6].

In this pilot study, we took advantage of obtaining peripheral blood mononuclear cells (PBMCs) in sufficient amount to carry out multiple investigations in a homogeneous series of adult patients who had chronic fatigue and diffuse myalgia developed after administration of Al oxyhydroxide (AH)-containing vaccines without other detected cause [12]. This condition, sometimes referred to as ASIA (autoimmune/inflammatory syndrome induced by adjuvants) [13,14], was historically identified by the detection in the deltoid muscle of an unusually longstanding histopathological lesion induced by AH particles and called macrophagic myofasciitis (MMF) [15].

A crucial role for individual susceptibility factors in reactions to AH adjuvants is very likely and supported by animal experiments [16]. According to the World Health Organization, MMF likely reflects the difficulty that some individuals have in clearing out the adjuvant from their body [17]. Admittedly, poor degradation of AH aggregates may represent a key factor of their inherent toxicity [18], and therefore, modalities of AH handling by immune cells deserves special attention.

AH adjuvants are nanoparticles forming stable micron-sized agglomerates. Together with adsorbed vaccine antigens they are handled as pathogen-like particles by innate immune cells, including avid capture, inflammatory response, antigen processing and subsequent presentation to generate adaptive immunity, and slow biodisposition [19,20]. Phagocytes involved in the process mainly derive from circulating inflammatory monocytes, likely attracted at the injection site by activated muscle-resident macrophages [21] in the setting of strong activation of the NALP3 inflammasome [22]. Electron microscopy has shown that some AH vaccine agglomerates ingested by macrophages are partially surrounded by an intracytoplasmic membrane [15,23], implying a lysosome-dependent intracellular degradation pathway in their cellular handling [24]. Two main clearance mechanisms may be at play: macro-autophagy that degrades intracellular components (hereafter referred to as autophagy); and also LC3-associated phagocytosis (LAP) [25], a form of phagocytosis that uses components of the autophagy pathway to eliminate pathogens, immune complexes, and extracellular debris [26]. Once ingested by cells, AH can alter phagolysosomal membrane integrity and impede autophagy degradative function [22,27], which may play a role in their adjuvant effect [28]. They may also alter structures and functions of mitochondria [29,30], an interesting feature if one considers that mitochondrial dysfunction being positively correlated with severity has been reported in ME/CFS [31].

In animal models, AH particles injected into muscle induce granulomas and translocate within immune cells to distant lymphoid organs, with subsequent low-rate accumulation in the brain and microglial activation [32,33,34,35]. In the same way, peripheral injections of Complete Freund Adjuvant (CFA) induce local granulomatous reactions with pain hypersensitivity [36] and neuropathologic inflammation [37], a well-known cause of central nervous system dysfunction [38]. Such mechanisms may underpin ME/CFS symptoms. They could be triggered by natural microbial adjuvants, i.e., the unique (non-mammalian) components of pathogens (e.g., CFA), or by inorganic materials with adjuvant activity (e.g., AH).

The aim of the present study was two-fold: (1) detecting specific AH- and AH vaccine (V)-handling modalities by macrophages of MMF patients, focusing on particle uptake and activation of both canonical and non-canonical autophagy pathways; (2) detecting immuno-metabolic effects of exposure on MMF cells relevant to clinical symptoms by thorough investigation of chemokine and cytokine release, mitochondrial metabolism, and oxidative stress markers. Four independent experiments were carried out in parallel, always including MMF and healthy control cells. Healthy cells’ responses have been previously published [20]. They were only used herein as a control to compare MMF cells’ responses against, the topic of the present study.

## 2. Materials and Methods

### 2.1. Patients and Controls

Written informed consent was obtained from all individuals included in the study in accordance with the Declaration of Helsinki recommendations. The protocols were approved by the ethic “Comité de Protection des Personnes” (IRB approval 2012, CPP Ile-de-France Paris 11). Patients were enrolled at the Henri Mondor Paris Est University Hospital, Créteil, France, and controls by Etablissement Français du Sang (EFS), affiliated to the hospital (# C CPSL UNT—N°18/EFS/033).

To reflect the fact that approximately 73% of those diagnosed with the MMF condition are women [39], patients were 8 genetic females (age: 58.1 ± 12.5 years) who had muscle biopsy-proven MMF according to previously detailed criteria [12]. Their clinical symptoms included chronic fatigue (8/8), widespread myalgia (8/8), and cognitive impairment (6/8). They had received 1 to 5 Al-adjuvanted vaccine (Alhydrogel^®^, https://www.invivogen.com/alhydrogel, accessed on 1 April 2022) shots within 10 years before biopsy (mean: 2.5; SD:1.4), with the last vaccine before onset of the first symptoms being against hepatitis B (n = 3), tetanus toxoid (n = 3), or hepatitis A (n = 2). The median time from symptoms to histological diagnosis of MMF was 3.5 years and median illness duration at time of blood sampling was 9.5 years. The CDC1994 criteria for ME/CFS were fully met in 5/8 patients, this ratio (62.5%) being consistent with the 47–71% range previously reported in MMF [12,40].

To cope with the difficulty in obtaining a sufficient number of control PBMCs for all experiments, healthy controls (gender- and age-matched) were obtained in two steps, corresponding to two experimental phases, i.e., (1) Al particle capture, western blots, and cytokines assay; and (2) mitochondrial metabolism evaluation and ROS generation assay. The first phase enrolled 11 female controls (age: 51.1 ± 6.6 years), and the second phase enrolled 10 female controls (age: 48.6 ± 10.2 years), with an overlap of 8 individuals between the two phases (Table A1 in Appendix A). No statistical difference for age was found in any experiment between MMF patients and controls (Table A1).

To further avoid bias due to age, correlation tests for age were systematically performed in both MMF and control groups. No significant correlation was found except for some decrease in GROα/CXCL1 in aged MMF patients, which was not found in the control group and had no impact on the global result (see Section 3.3). According to the available number of cells, 8 MMF patients (age: 58.1 ± 12.5 years) were used for internalization, inflammatory response, and autophagy evaluation, and 7 (age: 55.4 ± 10.7 years) for ROS production and mitochondrial metabolism, as a vial of cells was completely used during the first experimental phase (Table A1).

### 2.2. Blood Collection

Veinous blood was collected in an EDTA vacutainer tube and directly separated on Ficoll by centrifugation at 500 relative centrifugal force (RCF) for 20 min. PBMCs were washed with phosphate-buffered saline (PBS) and resuspended at a final concentration of 20.10^6^ cells per ml in freezing solution composed of 95% fetal bovine serum (FBS) and 5% of DiMethylSulfOxyde. Cell vials were frozen at −80 °C overnight and stored in liquid nitrogen until use.

### 2.3. Cell Culture

PBMCs were rapidly thawed at 37 °C in Dulbecco’s Modified Eagle’s Medium (DMEM, Fisher Scientific, 11966-025, Illkirch-Graffenstaden, France), centrifuged at 300 RCF for 5 min, and washed in PBS. After a second centrifugation, PBMCs were suspended in RPMI 1640 + Glutamax (Fisher Scientific, 61870-010) with 1% of penicillin/streptomycin (Fisher Scientific, 15140-122) and 1% of 200 mM L-glutamin (Fisher Scientific, 25030-024). Cells were plated for 3 h for adherence at a final density of 400 × 10^3^ cells per cm^2^ at 37 °C and 5% CO_2_ on different plates according to the analysis being performed. Ibidi coverslips were used for phagocytosis imaging (Ibidi, 80446, Gräfelfing, Germany), 24-well culture plates for cytokine assay and western blots (Costar, 3524, Corning, NY, USA), Seahorse XFe24 culture plates for mitochondrial metabolism tests (Agilent Technology, 102340-100, Massy, France), and 96-well culture plates for ROS assay (Dutscher, 353072, Corning-Falcon, Brumath, France). After 3 h of adherence, non-adherent cells were removed with the culture medium and fresh 37 °C differentiation medium composed of RPMI 1640 ATCC Modification (Fisher Scientific, A10491-01) with 1% of penicillin/streptomycin, 10% of FBS, and 0.1% of human macrophage colony-stimulating factor and granulocyte-macrophage colony-stimulating factor (Peprotech, 300-25 and 300-03, respectively, Neuilly-sur-Seine, France) was added for 6 days. After this differentiation time, half the volume of the medium culture was replaced for another 24 h by freshly prepared medium. Analyses were performed after a total of seven days of differentiation and exposure to AH particles and whole AH-adjuvanted EngerixB^®^ 20 vaccine (V) (GlaxoSmithKline, Rueil-Malmaison, France), which have been previously characterized by our lab in terms of size, charge, and shape [35,41].

### 2.4. Cell Imaging

Internalization of AH by PBMC-derived cells was monitored using Lumogallion fluorescent stain [42]. The AH–Lumogallion solution was produced by mixing vac-alu-250 (Invivogen) diluted at 5 mg Al/mL in RPMI 1640 ATCC Modification medium with 50 µM Lumogallion (Santa Cruz, sc-295368, Heidelberg, Germany). After overnight contact on a rocking table, pre-stained Al particles were centrifuged (10 min at 20,000 RCF) and resuspended in 1 mL of medium to obtain a final concentration of 5 mg Al/mL and stored at +4 °C until use. After seven days of differentiation, PBMCs were exposed for 4 h to Lumogallion-stained Al (50 µg Al/mL) and 45 min with Hoechst 33342 and LysoTracker green DND-26 (Life Technology SAS, H3570 and L7526, respectively, Courtaboeuf, France) according to the manufacturer’s recommendations to stain nucleus and lysosomes.

Cells were observed with a Zeiss Axio Observer Z1 microscope (63X objective) (Carl Zeiss S.A.S, Marly le Roi, France) under phase contrast and fluorescent imaging. Ten pictures per well were taken to visualize at least 50 cells per individual. Microphotographs were analyzed by the Icy software (V2.1.4.0 BioImage Analysis unit, Institut Pasteur, Paris, France) [43]. In each cell, the respective intensities of the Lumogallion and LysoTracker fluorescent signals were determined, and mean fluorescence intensity was calculated per µm^2^ of cell surface.

### 2.5. Biochemical Analyses

#### 2.5.1. Exposure to Al and Vaccine

Differentiated PBMCs were exposed for 4 h to the vehicle (PBS), or to AH (50 µg Al/mL), or to a whole vaccine (EngerixB^®^ 20, GSK) containing AH (500 µg AL/mL) diluted to obtain the same final concentration of 50 µg Al/mL.

#### 2.5.2. Western Blotting of Autophagy and LAP Components

Autophagy is a highly dynamic pathway, and therefore, steady-state measurements may give insufficient information. The turnover of key proteins must be artificially boosted or blocked in order to accurately estimate the autophagic flux. Thus, molecular aspects of Al handling were conventionally explored using western blotting with positive and negative controls provided by autophagy modulators, including the activator rapamycin (Rapa) at 100 nM and the inhibitor chloroquine (CQ) at 100 µM (Sigma Aldrich, R8781 and C66628, respectively, Merck, Darmstadt, Germany). Cells were exposed to the modulators 1 h before and then during the Al treatments. Autophagic function was characterized by changes in the expression of two key proteins, LC3 (microtubule-associated protein light chain 3) and p62 (also called SQSTM1), and LAP by its specific components Rubicon (run domain Beclin-1 interacting and cysteine-rich domain-containing protein) and Nox2 (NADPH oxidase 2). The number of autophagosomes is conventionally indicated by the amount of LC3, and especially its lipidated form LC3-II. LC3 conversion (LC3-I to LC3-II), assessed by the LC3 II/LC3 I ratio, indicates de novo autophagosome production [44]. p62 levels decrease when autophagolysosomes are successfully degraded at the end of the process, thus making p62 a marker of autophagic flux: when autophagy flux is blocked, p62 accumulates, while when autophagy is induced, p62 quantities decrease.

Analyses were performed on cell lysates produced by exposure to 100 µL of pre-heated TR3 solution after two washes on cold-PBS. The TR3 lysis solution is an aqueous solution of SDS at 170 mM (Sigma Aldrich, L-5750, Merck, Darmstadt, Germany), disodium phosphate at 10 mM (Sigma Aldrich, 1065761000), sodium orthovanadate at 1 mM (Sigma Aldrich, S6508), β-glycerophosphate at 10 mM (Sigma Aldrich, G9422), sodium pyrophosphate tetrabasic at 2.5 mM (Sigma Aldrich, P8010), sodium fluoride at 50 mM (Sigma Aldrich, 201154), 10% of glycerol (Sigma Aldrich, G6279), and cOmplete^TM^ Mini Protease Inhibitor cocktail (Sigma Aldrich, 11836153001) according to the manufacturer’s recommendations (Roche). Protein extracts were sonicated for 10 sec, then a BCA Pierce^TM^ protein assay kit (Fisher Scientific, 23225) was used to quantify the total amount of protein. For each individual, 14 µg of protein was subjected to electrophoresis on 4–12% Bis-Tris Mini Protein Gels (Invitrogen, NW04125BOX, Illkirch, France). The protein was then transferred to polyvinylidene difluoride membranes (Invitrogen, IB24002, Illkirch, France). The membranes were blocked in tris-buffered saline (150 mM NaCl, pH 8.0) containing 0.1% Tween 20 (TBST) and 2.5% cold-water fish skin gelatin (Sigma Aldrich, G7765, Merck, Darmstadt, Germany) for 60 min at room temperature with gentle agitation. Antibody staining was realized at +4 °C overnight with primary antibodies [rabbit anti-LC3B (1:1000; Life Technology, PA1-46286, Courtaboeuf, France), rabbit anti-SQSTM1/p62 (1:3000; Life Technology, PA5-20839, Courtaboeuf, France), rabbit anti Rubicon (1:1000; Ozyme, 8465S, Saint-Cyr l’école, France)]. Membranes were then washed for 30 min in TBST and incubated with a beta-actin HRP conjugate (1:10000; Santa Cruz, sc-47778HRP, Heidelberg, Germany), a Nox2 HRP conjugate (1:500; Clinisciences, ORB223721, Nanterre, France), or an HRP-conjugated anti-rabbit secondary antibody at room temperature for 1 h before a final wash of 30 min.

Signals were imaged using the SuperSignal West Femto substrate (Fisher Scientific, 34096, Illkirch, France) and Azure C600 (Azure Biosystems, Inc., Dublin, CA, USA). Protein bands were quantified by the ImageJ© software (V1.53i, Wayne Rasband and contributors, National Institute of Health, Bethesda, MA, USA) [45] and normalized to β-actin. It should be noted that considering the number of targeted proteins of the included individuals, and the electrophoretic gel size, it was not possible to perform a single-blot/-gel analysis. We thus chose to perform individual blots/gels with subsequent normalisation on beta-actin as a reference protein. 

#### 2.5.3. Cytokine/Chemokine Immunoassay

Release of cytokines/chemokines was determined after 4 h of exposure using a membrane antibody array based on the sandwich immunoassay principle. After 4 h of treatment, the culture medium was screened for interleukin (IL)-1β, IL-6, IL-8, IL-10, IL 12p40, IL-18, GROα/CXCL1, CCL2, CCL4, TGFβ, and TNF-α using specific membranes made by CliniSciences RayBio C-Series following the manufacturer’s recommendations. Imaging was performed by chemiluminescence using the manufacturer substrate. Cytokine/chemokine dots were quantified by the Icy software (V2.1.4.0 BioImage Analysis unit, Institut Pasteur, Paris, France) [43] and normalized using positive controls included in the membranes of the kit.

#### 2.5.4. Mitochondrial Metabolism Evaluation

Energy derived from nutrients is transformed via oxidative phosphorylation in adenosine triphosphate (ATP) by mitochondria. The electron transport chain located at the mitochondrial inner membrane expels protons from the mitochondrial matrix to the intermembrane space. These protons are driven back into the mitochondrial matrix through ATP synthase to generate ATP but also via proton leak pathways that do not generate ATP. ATP is the primary energy source for most biochemical and physiological processes, and mitochondria must constantly adapt to stress conditions to support cellular reactions and the higher energy level they need. On these grounds we used two tests for assessing mitochondrial function and reserve, namely, mitochondrial oxygen consumption rate (OCR) and spare respiratory capacity (SRC). However, OCR is only a partial proxy for energy metabolism due to the inherent variability in the amount of ATP generated per molecule of oxygen consumed by mitochondria. Since the rate of ATP generation is dependent on both the rate of oxygen consumption and the efficiency with which that consumed oxygen is used to make ATP, we determined ATP-linked OCR in parallel to basal and maximal OCR, as previously recommended [46]. Since coupling of ATP synthesis and substrate oxidation is not complete as protons can return to the matrix independently of ATP synthase, the proton leak rate was also determined for better insight into the energy metabolism. Finally, SRC, which is defined as the difference between basal ATP production and its maximal activity, was calculated. The SRC level determines the capacity or inability of cells to produce more ATP and overcome more stress [47].

After 7 days of differentiation, and following exposure to Al treatments, cells were washed and kept in Seahorse buffer during the measurements. Briefly, Seahorse buffer is an aqueous solution of EGTA (1 mM), MgCl_2_ (5 mM), KH_2_PO_4_ (10 mM), mannitol (220 mM), sucrose (70 mM), glutamate (10 mM), malate (2 mM), HEPES (2 mM), pyruvate (10 mM), BSA (0.2%), and ADP (2 mM). OCR was determined over 5 min increments. Three measures of stabilized OCR were taken before the sequential addition of the following compounds, each followed by three measurements of OCR: 10 mM of succinate (complex II substrate); 2.5 µM of oligomycin (ATP synthase inhibitor), 1 µM FCCP (an uncoupling protonophore), and 2.5 µM of antimycin A (complex III inhibitor). Immediately after the OCR was recorded, cells were lysed in 100 µL of 95 °C TR3 solution and the total amount of protein was measured using a BCA protein assay kit. The OCR measurements were standardized using the total protein amount in each well. The respiratory parameters were calculated as follows: basal respiration (succinate OCR-antimycin A OCR), ATP-linked respiration (succinate OCR-oligomycin OCR), proton leak rate (oligomycin OCR-antimycin A OCR), maximal respiration (FCCP OCR-antimycin A OCR), and SRC (FCCP OCR-succinate OCR).

#### 2.5.5. ROS Assay

ROS production was assessed using the fluorescent probe H_2_DCFDA for 45 min at 5 µM. The fluorescent signal was record on a plate reader (excitation: 485 nm, emission: 530 nm) along a 5 × 5 matrix grid for each well. Cells were then lysed by warm TR3 solution to measure the total amount of protein using a BCA protein assay kit. The ROS fluorescent signal was finally normalized by the individual amount of proteins. The ROS production of Al-treated cells was interpreted using the positive controls obtained by H_2_O_2_ treatment 45 min at 500 µM during ROS staining.

### 2.6. Statistical Analysis

All experimental data were analyzed by Jamovi V2.3.12 (Jamovi, Sydney, Australia) [48] and graphical presentations were built with GraphPad Prism V7(GraphPad, San Diego, CA, USA). According to the results of the Shapiro–Wilk test, evaluating normality of the distribution of data, we used either parametric or non-parametric tests for intra- and inter-group comparisons. Student’s t-test was used to compare the inter-group age distribution and fluorescence signal. The Mann–Whitney test was used to compare the results of western blotting, cytokine/chemokine assays, mitochondrial functional tests, and ROS production of MMF patients and controls. A Spearman test was applied to explore the presence of correlation between concerned variables and participant’s age.

A Friedman test followed by a Durbin–Conover pairwise post hoc test was performed to compare intra-group treatment effects.

All reported significance levels represent two-tailed *p*-values and critical alpha was set at 0.05 to indicate statistical significance.

## 3. Results

### 3.1. Immune Cells from MMF Patients Exhibit Similar Ability to Controls to Internalize Al Particles

**Figure 1:**
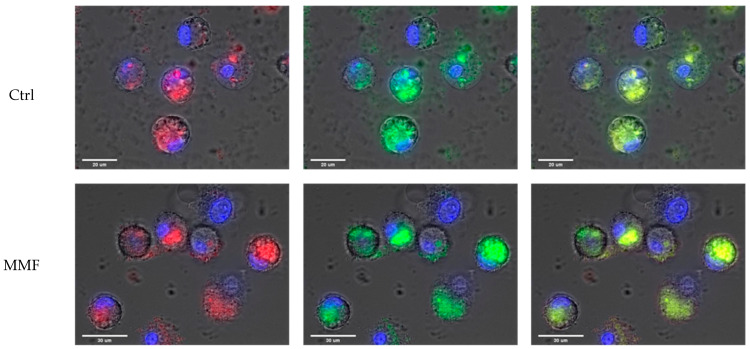
Example of observations of Al engulfment into control PBMCs (top panel) and MMF PBMCs (bottom panel) exposed for 4 h to Lumogallion-stained AH: Lumogallion signal in red (left panel), LysoTracker signal in green (central panel), and merged signals (right panel). Scale bars: control—20 µm; MMF—30 µm.

The cell size and morphology were quite similar in the controls and MMF patients after exposure. The specific fluorescence of Lumogallion was similarly detected within control and MMF cells after 4 h of treatment (Figure 1). The LysoTracker fluorescence was also similar in controls and MMF (Table A2). The MMF cells thus did not exhibit abnormal handling of Al particles during the first 4 h of contact (Table A2 and Figure 1). As previously noted in healthy individuals [17], intracellular detection of both Lumogallion and LysoTracker fluorescence points to the implication of lysosomes in reaction with Al particles, the clearance of which may therefore implicate either conventional autophagy of intracytoplasmic particles or an integrated LAP or a balance between the two pathways.

### 3.2. LAP Involvement and MMF Cell Limitations in Vaccine Clearance Mechanisms

**Figure 2:**
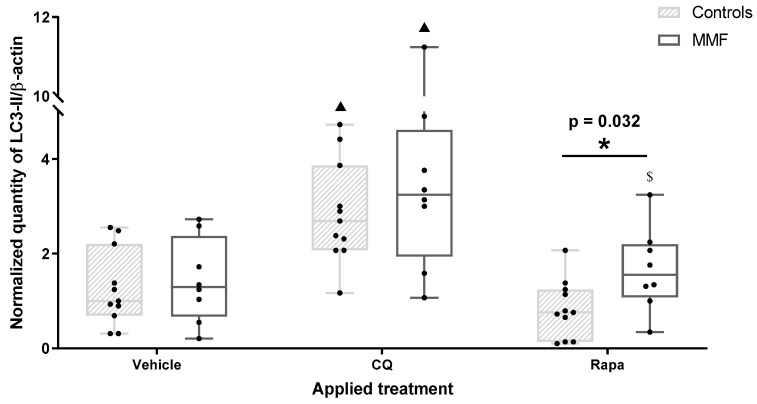
Expression level of LC3-II in differentiated PBMCs exposed for 4 h to several treatments. CQ: chloroquine; Rapa: rapamycin; * indicates statistical difference in Mann–Whitney test; ▲: *p* < 0.05 compared to vehicle treatment; $: *p* < 0.05 compared to CQ treatment.

Western blots of autophagy proteins were interpreted according to Klionsky’s recommendations [44]. Autophagy modulator effects were investigated first on PBMCs not exposed to Al compounds. CQ is known to cause both initial autophagy stimulation and blockade of the delivery of sequestered cargo to the lysosomes [49,50]. Consistently, both control and MMF cells treated by CQ showed an increase in both LC3-II and LC3-II/LC3-I without significant variation in p62, demonstrating autophagosome accumulation without proportionate degradation (Table A3). The activator (Rapa) induced somewhat different effects in the controls and MMF patients. In the controls, Rapa induced a decrease in both LC3-II and p62, as expected, demonstrating an efficient autophagic flux, whereas MMF cells treated by the autophagy activator showed an increase in the LC3-II/LC3-I ratio compared to the vehicle (Table A3), and more LC3-II than controls (Figure 2). This suggested a natural propensity of MMF cells to activate an additional LC3-dependant pathway such as LAP, which Rapa can also help to mature [51]. PBMCs were then exposed to AH or whole vaccine with or without autophagy modulators.

AH alone increased LC3-II and reduced p62 compared to the vehicle in both MMF and control cells, demonstrating autophagic flux activation by AH. As observed after Rapa stimulation, AH induced an increase in the LC3-II/LC3-I ratio that was restricted to MMF cells (Table 1 and Table A3); AH alleviated CQ-induced autophagic flux inhibition, since, compared to CQ alone, AH + CQ reduced the p62 level in both MMF and control cells (Table A3). 

However, unlike the controls, MMF cells neither showed an increase in LC3-II nor the LC3-II/LC3-I ratio under AH + CQ, possibly revealing an interfering phenomenon blurring the results in MMF cells. AH added its own autophagy activation effects to those of Rapa, since the p62 decrease was more pronounced with AH + Rapa than with AH alone and Rapa alone, in both MMF and control cells (Table A3).

The whole vaccine (V) had similar effects to AH on the MMF cells, including autophagic flux activation, assessed by the LC3-II/LC3-I ratio increase and p62 decrease, and both alleviation of CQ inhibition effects and strengthening of Rapa activation effects, assessed by the p62 autophagic flux marker (Table 1 and Table A3). In the control cells, V induced a similar p62 decrease but a weaker increase in LC3-II than AH (Table 1). As observed with AH, there were differences in LC3-II expression and the LC3-II/LC3-I ratio in the MMF cells compared to the control cells after V treatment, seemingly pointing to an alternative LC3 production, possibly implying that there is a competitive pathway that could interfere with the canonical autophagy pathway.

At this stage, we determined the levels of Rubicon and Nox2, two proteins uniquely required for LAP [44], which is utilized by phagocytes to kill and digest extracellular pathogens. LAP is initiated at the cell surface by receptors that recruit elements of the autophagy machinery, like LC3, to the phagosome. Rubicon, which is involved in LAP, also represents one of the few negative regulators of conventional autophagy, whereas Nox2 plays a central role in pathogen killing through ROS production in the phagosome lumen [25,52,53]. Autophagy modulators had no effect on the amount of Rubicon and Nox2, as expected for proteins unrelated to canonical autophagy (Table A3). 

**Figure 3:**
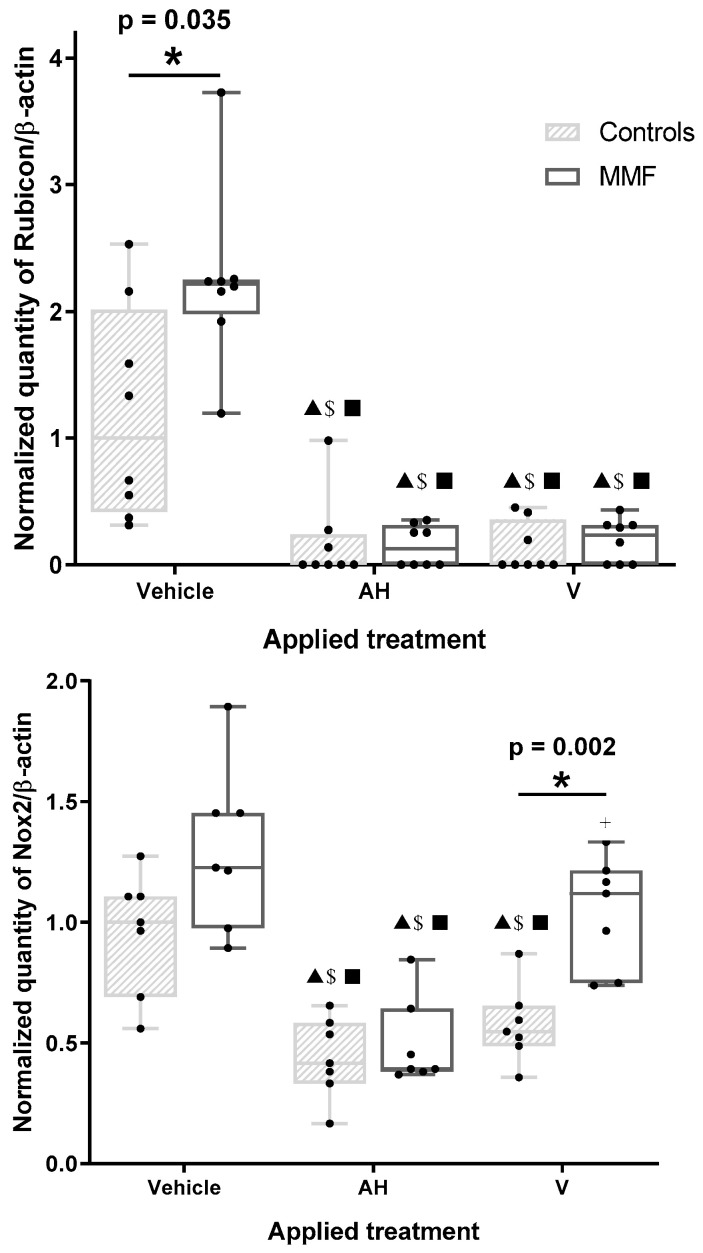
Expression level of Rubicon (upper panel) and Nox2 (lower panel) in differentiated PBMCs exposed for 4 h to several treatments. AH: aluminum oxyhydroxide (Alhydrogel^®^); V: whole vaccine (EngerixB^®^ 20). * indicates statistical differences in Mann–Whitney test; ▲: *p* < 0.05 compared to vehicle treatment; $: *p* < 0.05 compared to CQ treatment; ■: *p* < 0.05 compared to Rapa treatment; +: *p* < 0.05 compared to AH treatment.

The amount of Rubicon in MMF cells unexposed to AH or V was markedly greater than in the controls (Figure 3 and Table A3), suggesting the MMF cells had an enhanced LAP capacity, with a possible propensity to produce more LC3 protein than the controls by adding this source of LC3 to the canonical autophagy. AH and V decreased Rubicon in both the control and MMF cells (Figure 3). This decrease was unaffected by addition of CQ or Rapa (Table A3). 

Similarly to Rubicon, the Nox2 level was decreased by AH and V in the control cells. A Nox2 decrease was induced by AH in the MMF cells, but this did not occur with V, leading to higher levels of Nox2 in the MMF cells compared to the control cells in this condition (Figure 3 and Table A3). Nox2 was reduced, however, in MMF cells after V + Rapa treatment compared to Rapa alone. Addition of CQ had no effect on Nox2 responses.

The observed reduction in both Rubicon and Nox2 levels strongly suggests consumption of these molecules in the presence of AH, and thus, implicates LAP in the capture and disposition of the adjuvant by phagocytic cells. Why, unlike for the controls, the MMF cells’ Nox2 response to the vaccine differed from that observed with the adjuvant alone, and did not parallel the Rubicon response, remains unclear and deserves kinetic evaluation over longer exposures.

Autophagy and LAP are two distinct pathways, sharing some autophagy-related proteins in their machinery [53], that clear out differently located targets. Autophagy clears intracytoplasmic compounds, such as defective proteins and organelles, whereas LAP clears extracellular compounds, such as pathogens or dead cells [53]. Consistently, LAP seems to be clearly involved in the capture and handling of both AH and vaccine particles by healthy PBMCs [20]. Canonical autophagy may, in addition, clears membrane-free intracytoplasmic AH and vaccine aggregates, which are frequently observed in macrophages, presumably because of direct membrane damage by AH [15]. Interestingly, LAP may be more easily induced in MMF phagocytes which express a greater amount of Rubicon. However, MMF cells possibly exhibit some defect in using Nox2 when exposed to whole vaccine, which may indicate that something is going wrong in the last steps of vaccine handling by LAP in these individuals. Nevertheless, Rubicon itself acts both as a negative regulator of canonical autophagy and a key modulator of the inflammatory response [54]. Rubicon upregulation in MMF cells may, therefore, impede autophagic clearance of the free, i.e., membrane-unbound, cytoplasmic Al–vaccine agglomerates that are abundantly observed by electron microscopy in MMF macrophages [15] and cause immunosuppression (see Section 3.3).

### 3.3. MMF Cells Produce More Pain-Inducing CXC Chemokines and Less TNF-α Than Controls

**Figure 4:**
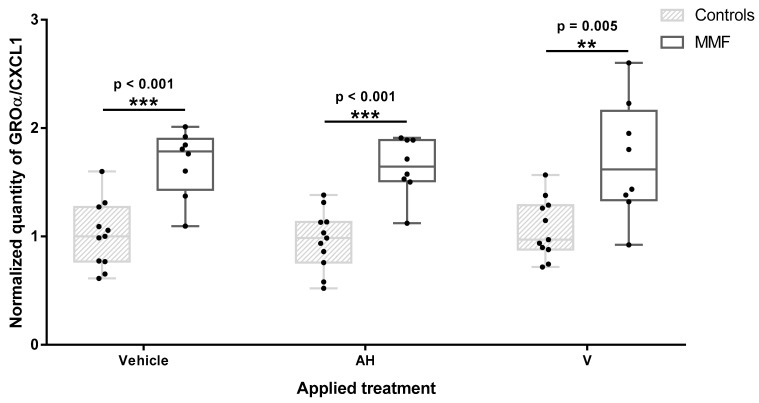
Level of GROα/CXCL1 in MMF patients’ and controls’ differentiated PBMCs exposed for 4 h to Al-containing treatments. * indicates statistical differences in Mann–Whitney test (**: *p* < 0.01; ***: *p* < 0.001); AH: aluminum oxyhydroxide (Alhydrogel^®^); V: whole vaccine (EngerixB^®^ 20).

Among the 11 cytokines and chemokines screened in the supernatants after 4 h of treatment, 4 were below the detection threshold (IL-1β, IL-12p40, IL-18, and TGF-β). Three chemokines (GROα/CXCL1, IL8/CXCL8, and MCP1/CCL2) were produced at high levels in at least one experimental condition. MMF cells systematically released higher level of GROα/CXCL1 than healthy controls after vehicle, adjuvant, and vaccine exposure (Figure 4 and Table A4). GROα/CXCL1 levels decreased with age in MMF patients as assessed by the Spearman test, but the detected levels always remained higher in the MMF patients (mean age 58 yrs) than in the healthy controls (mean age 51yrs). Adjuvant and vaccine treatments had no influence on this chemokine release, indicating that high GROα/CXCL1 expression is a basal characteristic of MMF cells. The IL-8, also named CXCL8, level was also higher in MMF patients than in the controls after adjuvant treatment. In contrast, MCP1/CCL2 release was significantly increased only in the healthy controls after vaccine exposure. 

GROα/CXCL1 and IL-8/CXCL8 are two CXC class chemokines that both attract neutrophils to sites of inflammation and are involved in pain mediation [55,56]. Indeed, chemokines increase pain sensitivity by acting on their receptors expressed along the pathway of pain in the peripheral nerves, the dorsal root ganglia, and the spinal cord [57]. GROα/CXCL1 and IL-8/CXCL8 upregulations have been repeatedly documented in ME/CFS and fibromyalgia, two painful and largely overlapping conditions. GROα/CXCL1, which mediates both inflammatory and neuropathic pain, has been detected at increased levels in the blood of ME/CFS patients [58] and found to correlate with the severity of the disease [59]. As in the present study, increased GROα/CXCL1 production by monocytes has been found in fibromyalgic patients compared to healthy controls, this increase being observed at rest and after monocyte activation by lipopolysaccharide [60]. IL8/CXCL8 is another circulating pain marker reportedly increased in ME/CFS, correlated with disease severity [61] and more elevated in recently ill patients. In the same way, IL8/CXCL8 is one of the most constantly reported inflammatory molecule increased in fibromyalgia and is correlated with the severity of symptoms [62,63,64,65,66,67]. 

In contrast, to CXC chemokines, MCP1/CCL2, another chronic-pain-inducing chemokine [68], was not upregulated in MMF cells in our short-term experiment. This was surprising since selective elevation of MCP1/CCL2 serum levels have been previously reported in MMF patients [69], as well as in ME/CFS and fibromyalgia patients [70]. In contrast, we observed increased release of MCP1/CCL2 by healthy PBMCs exposed to adjuvanted vaccine. This was not observed with the adjuvant alone, pointing to a critical role of the vaccine antigen (i.e., a specific or more pronounced effect than the adjuvant) [20]. The observed effect of the vaccine, however, was consistent with the known role of MCP1/CCL2 in the attraction of circulating inflammatory monocytes and dendritic cells to the muscle [18], in chronic granuloma formation [71], and in the development of polarized Th2 immune responses [72] that typically result from intramuscular injection of Al-containing vaccines. The MCP1/CCL2 response of the MMF cells to the vaccine may deserve longer kinetic studies to be fully appreciated.

**Figure 5:**
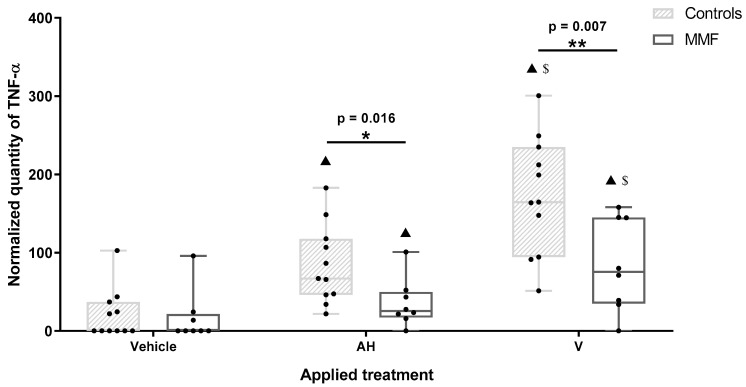
Expression level of TNF-α in MMF patients and controls’ differentiated PBMCs exposed for 4 h to Al-containing treatments. * indicates statistical differences in Mann–Whitney test (*: *p* < 0.05; **: *p* < 0.01); ▲: *p* < 0.001 compared to vehicle treatment; $: *p* < 0.001 compared to AH treatment; AH: aluminum oxyhydroxide (Alhydrogel^®^); V: whole vaccine (EngerixB^®^ 20).

TNF-α was the most importantly released cytokine after 4 h of treatment. TNF-α release was increased by both AH and V, in both the MMF and healthy control groups. However, TNF-α release increased more after V than AH treatment (Figure 5 and Table A4), pointing to a supplementary antigen effect. The MMF cells expressed a pattern of TNF-α response similar to the controls but at significantly lower levels in the presence of both AH alone and V (Figure 5 and Table A4). The weak TNF-α responses of the MMF cells were consistent with high expression of the sentinel Rubicon that downplays inflammation [54]. More generally, the downregulation of inflammatory molecules previously documented in both MMF [69] and ME/CFS [11,70,73], has been usually interpreted as an immune system “burnout” following an inappropriately long-lasting immune stimulation [11], preventing restoration of the initial status [10]. It is possible, however, that poor ability to mount an inflammatory response in ME/CFS reflects a more generalized protective stress-induced hypometabolic state resembling hibernation, minimizing energy consumption, that has been well characterized and described as “dauer” in *Caenorhabditis elegans* [74].

To summarize, PBMCs of patients with MMF released higher amounts of the pain-inducing chemokines GROα/CXCL1 and IL8/CXCL8 and lower amounts of TNF-α than healthy individuals after exposure to Al particles with or without antigens.

### 3.4. MMF Cells Exposed to Al Particles with or without Antigens Show Exacerbated Oxygen Consumption, Limited Spare Respiratory Capacity, and Increased Proton Leak Weakening Energy Production

**Figure 6:**
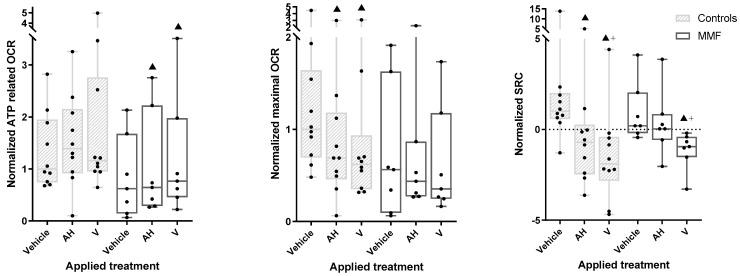
ATP-related OCR (**left** panel), maximal OCR (**central** panel), and SCR (**right** panel) in MMF patients’ and controls’ differentiated PBMCs exposed for 4 h to Al-containing treatments. ▲: *p* < 0.05 compared to vehicle treatment from same group; +: *p* < 0.05 compared to AH treatment; AH: aluminum oxyhydroxide (Alhydrogel^®^); V: whole vaccine (EngerixB^®^ 20).

As a general trend, the OCR tended to be lower in MMF cells compared to the controls (e.g., 56% of controls had max OCR under vehicle exposure). Though it did not reach the significance threshold in our statistical tests, this could possibly suggest some limitation of aerobic metabolism in MMF cells (Figure 6—central panel and Table A5). However, this was not associated with a limited capacity of MMF cells to increase their basal-state OCR indices upon exposure to the adjuvant and vaccine. 

The MMF cells, unlike the controls, showed a significant increase in basal OCR after vaccine treatment compared to the vehicle. The MMF cells also showed an increased ATP-linked OCR after the vaccine and adjuvant treatments (Figure 6—left panel and Table A5), suggesting systemic adaptation of the MMF cells to vaccine stress, presumably based on mitochondrial fusion/fission dynamics [75].

Despite the observed vaccine-induced OCR increase, the MMF cells showed a significant decrease in SRC and significant increase in proton leak after exposure to the vaccine compared to the vehicle (Figure 6—right panel and Table A5). An SRC reduction was also observed in controls after exposure to the vaccine and, to a lesser degree, the adjuvant, but without a significant increase in proton leak.

Taken together, these results indicate that MMF mitochondrial metabolism exhibits exacerbated reaction to adjuvanted vaccine, leading to prompt SRC saturation associated with marked proton leak, reflecting poor coupling between proton production and ATP generation by the respiratory chain.

### 3.5. MMF Cells Produce Little ROS after Short Adjuvant and Vaccine Exposures

ROS identification by the fluorescent probe H_2_DCFDA was first tested in PBMCs exposed for 4 h to H_2_O_2_. In this positive control study, ROS production increased in both healthy control and MMF cells. Compared to the vehicle, vaccine exposure (4 h) produced a small but statistically significant increase in ROS in the control but not in the MMF cells. ROS did not increase after AH exposure (Table A6). Usually, for mononuclear cells such as dendritic cells, ROS production is slower but sustained after immune stimulation compared to neutrophils [76], possibly explaining the poor ROS induction in our experiments by inappropriately short exposure. In the presence of H_2_O_2_, AH alone and V induced significantly more ROS, but surprisingly, this ROS overproduction remained lower than the positive control alone. Adsorption of H_2_O_2_ by AH possibly limited its oxidative capacity, as previously reported [77].

In brief, in our experimental conditions, MMF and control cells exhibited limited oxidative stress and behaved quite similarly in term of ROS production (Table A6). Though seemingly inconsistent with classical views on the role of oxidative stress in ME/CFS, these results are in line with those of a clinical investigation in MMF patients showing a decrease in anti-oxidant molecules (glutathion peroxidase, vitamin E, selenium) without an increase in oxidative stress markers in the blood [78].

## 4. Conclusions

**Figure 7:**
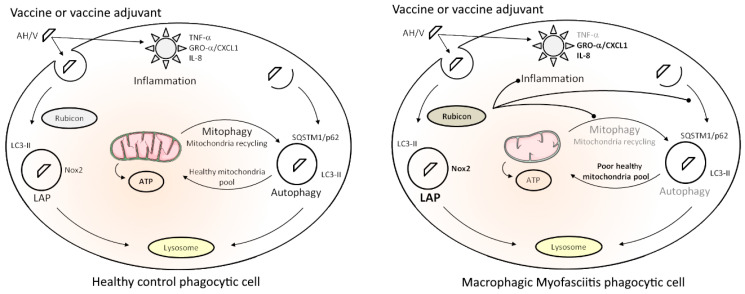
Synthetic scheme of healthy immune cell responses to immune stimulation (**left**) and specificities of immune cells from MMF patients (**right**). See the Section 4 for details about the represented mechanisms in both situations. “
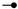
” represents the three main consequences expected of Rubicon overexpression in MMF phagocytic cells.

Our data indicate that AH vaccine particles are quickly recognized and internalized by innate immune cells and activate a double clearance mechanism, LAP and canonical autophagy, thus behaving as pseudo-pathogens that are handled by immune cells as both extracellular invaders (LAP) and intracytoplasmic aliens (autophagy). We previously showed that Al oxyhydroxide particles induce an inflammatory response starting with TNF-α release in healthy PBMCs, the response being enhanced by adsorbed vaccine antigens [20]. We showed herein that TNF-α responses to the same exposure are weaker in MMF cells and are associated with intrinsically high expression of Rubicon, a major LAP protein known to downplay inflammatory responses and to inhibit autophagy of intracytoplasmic particles. MMF cells also released increased amounts of CXC chemokines, known to induce pain. Mitochondrial metabolism that constantly adapts ATP production to energy-requiring functions, such as particle recognition, phagocytosis, and clearance mechanisms, was deregulated in MMF cells. MMF cells exposed to the Al-adjuvanted vaccine showed a burst of oxygen consumption, contrasting with suboptimal maximal oxygen consumption, limited spare respiratory capacity, and increased uncoupling with ATP production due to proton leak, thus predicting the reduced ability of mitochondria to adapt their energy production to future cell needs (Figure 7). This is reminiscent of what has been reported in ME/CFS [31] and may play a crucial role in chronic fatigue. To the best of our knowledge, this exploratory study is the first one that clearly demonstrates specificities of immune cells from MMF patients reacting to an inflammatory stimulus such as AH or an AH-containing vaccine. Thus, taken together, the present findings provide a plausible explanation for the cardinal clinical features, i.e., widespread myalgia and chronic fatigue, typically associated with long-term biopersistence of adjuvanted vaccine particles within immune cells, which is the hallmark of MMF [6]. In light of the critical roles of mitochondria, well beyond the traditional task of cellular energetic supply, such as acting as an environmental sensor to a large panel of stimuli and dynamic information integration [79], mitochondria should be considered as a key point for future experiments.

The present study suffered from three main limitations: (1) the relatively low number of tested patients (n = 8); (2) the short-term evaluation of exposure effects; (3) the use of a single AH or V challenge, when the disease typically manifests after multiple vaccinations. Such limitations are not unusual in pilot multi-task studies of rare chronic diseases. It would have been optimal, but unrealistic, to obtain PBMCs of the same individuals before and after the onset of clinical symptoms. 

The perspectives of our study are as follows. Additional studies with a larger cohort of patients, further investigations on the role of LAP in vaccine clearance, and evaluation of long-term vaccine exposure effects on inflammatory response and mitochondrial metabolism would be welcomed to confirm our results and translate them into clinical benefits for patients. These benefits could include (1) delineation of individual profiles of patients at risk of developing intolerance to AH vaccines or other particulate triggers of ME/CFS; and (2) development of pharmacological interventions aimed at favoring biodisposition of AH particles by targeting autophagic pathways.

We believe that investigating such research avenues will maintain and improve public trust in vaccination and adjuvants by (1) supporting patients in their diagnosis and care; (2) allowing detection of high-risk populations; and (3) helping to produce cellular and/or animal models to test adapted drugs. More generally, it seems likely that advances in these topics in MMF may offer a great opportunity to develop appropriate precision medicine for ME/CFS of other causes.

## Figures and Tables

**Table 1:** Autophagy-/LAP-related protein quantification in MMF patients and controls’ differentiated PBMCs exposed for 4 h to several treatments.

Treatment	Group	Vehicle	AH	V
LC3-II	Control	1.00 (0.79–1.79)	2.24 (1.81–3.33) ▲	1.24 (1.03–1.52) +
	MMF	1.29 (0.91–1.94)	2.10 (1.67–3.52) ▲	1.71 (1.40–2.89)
LC3-II/LC3-I	Control	1.00 (0.66–1.33)	1.21 (1.12–1.59)	1.12 (0.98–1.33)
	MMF	0.66 (0.50–0.95)	1.42 (1.20–1.48) ▲	1.44 (1.17–1.70) ▲
SQSTM1/p62	Control	1.00 (0.55–1.57)	0.60 (0.39–0.72) ▲	0.58 (0.42–0.78) ▲
	MMF	1.33 (0.93–1.61)	0.64 (0.53–0.77) ▲	0.71 (0.59–0.81) ▲

Results are expressed as median and quartiles (in brackets) of protein/b-actin level normalized by vehicle median. AH: aluminum oxyhydroxide; V: whole vaccine (EngerixB^®^ 20); ▲: *p* < 0.05 compared to vehicle treatment; +: *p* < 0.05 compared to AH treatment.

## Data Availability

The datasets used and/or analyzed during the current study are available from the corresponding author after reasonable request.

## Abbreviations

AH	Aluminum oxyhydroxide, commercially available as Alhydrogel^®^
Al	Aluminum
ASIA	Autoimmune/inflammatory syndrome induced by adjuvants
ATP	Adenosine triphosphate
CFA	Complete Freund Adjuvant
CQ	Chloroquine
FBS	Fetal bovine serum
IL	Interleukin
LAP	LC3-associated phagocytosis
LC3	Microtubule-associated protein light chain 3
ME/CFS	Myalgic encephalomyelitis/chronic fatigue syndrome
MMF	Macrophagic myofasciitis
Nox2	NADPH oxidase 2
OCR	Oxygen consumption rate
PBMC	Peripheral blood mononuclear cell
PBS	Phosphate-buffered saline
Rapa	Rapamycin
RCF	Relative centrifugal force
ROS	Reactive oxygen species
Rubicon	Run domain Beclin-1-interacting and cysteine-rich domain-containing protein
SRC	Spare respiratory capacity
TBST	Tris-buffered saline with 0.1% Tween 20
V	Whole Al-based vaccine (EngerixB^®^ 20)

## Appendix A

**Table A1:** Age and number of individuals according to the considered experiment.

Experiments	Group	N	Minimum	Mean ± SD	Maximum	*t*-Test		
						t	df	*p*
Al engulfment observation Western blots Cytokine assay	Control	11	42	51.09 ± 6.58	65	1.60	17	n.s.
	MMF	8	37	58.13 ± 12.52	77			
ROS assay Mitochondrial metabolism evaluation	Control	10	35	48.60 ± 10.16	65	1.33	15	n.s.
	MMF	7	37	55.43 ± 10.72	69			

n.s.: not significant.

**Table A2:** Cell size and Lumogallion and LysoTracker fluorescences observed after four hours of treatment.

Group	Control	MMF	*t*-Test		
			t	df	*p*
Minimum cell size (µm^2^)	202.03 ± 41.78	198.80 ± 28.49	0.19	16	n.s.
Mean cell size (µm^2^)	366.90 ± 65.40	364.00 ± 88.98	0.08	16	n.s.
Max cell size (µm^2^)	652.40 ± 166.50	704.20 ± 229.90	0.55	16	n.s.
LysoTracker fluorescence/µm^2^ (a.u.)	1.00 ± 0.48	1.03 ± 0.29	0.13	16	n.s.
Lumogallion fluorescence/µm^2^ (a.u.)	1.00 ± 0.52	0.99 ± 0.28	0.04	16	n.s.

Results are expressed as mean ± SD; a.u.: arbitrary unit corresponding to the average of pixel value by µm^2^ normalized by controls’ mean; n.s.: not significant.

**Table A3:** Autophagy-/LAP-related protein quantification in MMF patients’ and controls’ differentiated PBMCs exposed for 4 h to several treatments.

Treatment		Vehicle		CQ	Rapa		AH	AH + CQ	AH + Rapa	V		V + CQ		V + Rapa		Friedman	
	Group															Χ²	*p*
LC3–II	Control	1.00 (0.79–1.79)		2.69 (2.19–3.43) ▲	**0.76** **(0.40–1.19)** **▲ $**	*****	2.24 (1.81–3.33) ▲ ■	4.14 (3.14–4.95) $ +	1.66 (1.28–2.28) ■ +	1.24 (1.03–1.52) $ ■ +		3.24 (2.52–3.53) ♦		1.55 (0.98–2.00) ■		**57.36**	**<0.001**
	MMF	1.29 (0.91–1.94)		3.24 (2.65–4.04) ▲	**1.55** **(1.23–2.11)** **$**		2.10 (1.67–3.52) ▲ ■	3.74 (2.90–4.35)	1.76 (1.27–2.02)	1.71 (1.40–2.89)		3.43 (3.29–3.77) ♦		1.22 (1.02–2.18)		**27.73**	**<0.001**
LC3–II /LC3–I	Control	1.00 (0.66–1.33)		1.37 (0.96–1.90) ▲	0.89 (0.55–1.16) $		1.21 (1.12–1.59) ■	1.60 (1.26–1.93) +	1.70 (1.10–2.17) ■	1.12 (0.98–1.33) $		1.58 (1.27–1.80) ♦		1.47 (1.05–1.58) ■ ♦		**33.83**	**<0.001**
	MMF	0.66 (0.50–0.95)		1.43 (1.25–1.63) ▲	1.29 (1.23–1.70) ▲		1.42 (1.20–1.48) ▲	1.82 (1.05–2.14)	1.92 (1.54–2.42)	1.44 (1.17–1.70) ▲		1.95 (1.21–2.39)		1.47 (1.20–2.15)		**21.17**	**0.007**
SQSTM1/ p62	Control	1.00 (0.55–1.57)		1.06 (0.88–1.31)	0.60 (0.48–0.77) ▲ $		0.60 (0.39–0.72) ▲ $	0.66 (0.57–0.87) $	0.33 (0.19–0.40) ■ +	0.58 (0.42–0.78) ▲ $		0.63 (0.53–0.84) $		0.21 (0.19–0.38) ■ ♦		**48.28**	**<0.001**
	MMF	1.33 (0.93–1.61)		1.27 (1.16–1.36)	0.83 (0.61–1.02) $		0.64 (0.53–0.77) ▲ $	0.86 (0.60–1.07) $	0.37 (0.29–0.51) ■ +	0.71 (0.59–0.81) ▲ $		0.85 (0.76–0.90) $		0.48 (0.42–0.50) ■ ♦		**36.13**	**<0.001**
Rubicon	Control	**1.00** **(0.50–1.73)**	*	1.18 (0.76–2.26)	1.95 (1.37–2.42)		0.00 (0.00–0.17) ▲ $ ■	0.00 (0.00–0.24) $	0.00 (0.00–0.19) ■	0.00 (0.00–0.25) ▲ $ ■		0.00 (0.00–0.22) $		0.00 (0.00–0.18) ■		**51.77**	**<0.001**
	MMF	**2.22** **(2.10–2.24)**		2.10 (2.02–2.46)	2.22 (2.15–2.64)		0.13 (0.00–0.27) ▲ $ ■	0.16 (0.00–0.34) $	0.10 (0.00–0.27) ■	0.24 (0.00–0.31) ▲ $ ■		0.25 (0.00–0.44) $		0.23 (0.00–0.30) ■		**50.61**	**<0.001**
NOX2	Control	1.00(0.83–1.11)		0.93 (0.88–1.05)	1.04 (0.81–1.09)		0.42 (0.36–0.56) ▲ $ ■	0.35 (0.28–0.48) $	0.35 (0.30–0.62) ■	**0.55** **(0.51–0.63)** **▲ $ ■**	*	**0.62** **(0.55–0.65)** **$**	*	**0.62** **(0.46–0.71)** **■**	#	**37.78**	**<0.001**
	MMF	1.23 (1.10–1.45)		1.18 (1.09–1.21)	1.17 (0.89–1.27)		0.39 (0.39–0.55) ▲ $ ■	0.50 (0.36–0.68) $	0.58 (0.40–0.71) ■	**1.12** **(0.86–1.19)** **+**		**0.85** **(0.74–1.13)**		**0.81** **(0.70–1.14)** **■**		**37.46**	**<0.001**

Results are expressed as median and quartiles (in brackets) of protein/b-actin level normalized by vehicle median. CQ: chloroquine; Rapa: rapamycin; AH: aluminum oxyhydroxide; V: whole vaccine (EngerixB^®^ 20); *: *p* < 0.05 in Mann–Whitney test between control and MMF groups for treatment indicated in column. #: *p* = 0.055 in Mann–Whitney test between control and MMF groups for treatment indicated in column; ▲: *p* < 0.05 compared to vehicle treatment. $: *p* < 0.05 compared to CQ treatment; ■: *p* < 0.05 compared to Rapa treatment; +: *p* < 0.05 compared to AH treatment; ♦: *p* < 0.05 compared to V treatment.

**Table A4:** Cytokine quantification in MMF patients’ and controls’ differentiated PBMCs exposed for 4 h to Al-containing treatments.

Treatment		Vehicle		AH		V		Friedman	
	Group							Χ^2^	sig.
IL-1b	Control	Only three samples above the detection limit. Statistics not applied.							
	MMF								
IL-6	Control	1.00 (0.48–2.29)		1.65 (0.77–1.94)		1.97 (1.08–2.61)		5.09	n.s.
	MMF	1.88 (1.07–2.72)		2.15 (1.56–2.63)		2.62 (1.89–3.08)		1.75	n.s.
IL-8	Control	1.00 (0.79–1.10)		**0.93** **(0.85–1.03)**	*****	1.09 (1.01–1.18)		5.64	n.s.
	MMF	1.07 (0.98–1.11)		**1.08** **(1.02–1.19)**		1.14 (1.07–1.26)		4.00	n.s.
IL-10	Control	1.00 (0.23–1.32)		0.62 (0.00–2.222)		2.47 (0.53–3.32)		3.35	n.s.
	MMF	1.67 (0.82–2.19)		0.75 (0.00–2.15)		1.00 (0.00–1.78)		0.67	n.s.
IL-12p40	Control	No sample above the detection limit. Statistics not applied.							
	MMF								
IL-18	Control	Only four samples above the detection limit. Statistics not applied.							
	MMF								
GROa/CXCL1	Control	**1.00** **(0.77–1.18)**	*****	**0.99** **(0.81–1.13)**	*****	**0.97** **(0.89–1.27)**	*****	4.55	n.s.
	MMF	**1.78** **(1.55–1.86)**		**1.65** **(1.52–1.89)**		**1.62** **(1.37–2.02)**		1.75	n.s.
CCL2	Control	1.00 (0.95–1.14)		1.05 (0.98–1.15)		1.14 (1.08–1.21) ▲ $		7.09	0.029
	MMF	1.07 (0.96–1.16)		1.14 (1.11–1.20)		1.12 (1.05–1.33)		5.25	n.s.
CCL4	Control	1.00 (0.71–1.17)		1.05 (0.75–1.16)		1.11 (0.82–1.30)		1.27	n.s.
	MMF	0.85 (0.82–0.91)		0.84 (0.81–0.94)		0.86 (0.80–0.91)		1.00	n.s.
TGF-b	Control	No sample above the detection limit. Statistics not applied.							
	MMF								
TNF-a	Control	0 (0–30.84)		**67.07** **(46.7–112.22)** **▲**	*****	**164.62** **(121.05–223.65)** **▲ $**	*****	**20.18**	**<0.001**
	MMF	0 (0–16.43)		**25.53** **(20.2–45.53)** **▲**		**75.54** **(37.69–144.75)** **▲ $**		**14.00**	**0.001**

Results are expressed as median and quartiles (in brackets) of cytokine pixel signal/internal positive control. When possible, results were normalized by vehicle median. AH: aluminum oxyhydroxide (Alhydrogel^®^); V: whole vaccine (EngerixB^®^ 20). n.s.: not significant. *: *p* < 0.05 in Mann–Whitney test between control and MMF groups; ▲: *p* < 0.05 compared to vehicle treatment; $: *p* < 0.05 compared to AH treatment.

**Table A5:** Mitochondrial parameters in MMF patients’ and controls’ differentiated PBMCs exposed for 4 h to Al-containing treatments.

Treatment		Vehicle	AH		V	Friedman	
	Group					Χ²	*p*
Basal OCR	Control	1.00 (0.76–1.88)	1.30 (0.87–1.67)		1.14 (1.02–1.65)	1.40	n.s.
	MMF	0.59 (0.30–1.25)	0.72 (0.37–1.15)		0.75 (0.46–1.48) ▲ +	**7.92**	**0.019**
ATP related OCR	Control	1.00 (0.80–1.79)	1.39 (1.01–1.99)		1.16 (0.98–2.20)	2.89	n.s.
	MMF	0.62 (0.26–1.29)	0.64 (0.36–1.48) ▲		0.77 (0.54–1.44) ▲	**8.86**	**0.012**
Max OCR	Control	1.00 (0.77–1.46)	0.69 (0.51–1.04) ▲		0.62 (0.41–0.70) ▲	**15.00**	**<0.001**
	MMF	0.56 (0.22–1.11)	0.44 (0.29–0.70)		0.35 (0.26–0.84)	3.43	n.s.
Proton leak	Control	1.00 (0.79–1.97)	**1.27** **(0.91–1.50)**	*****	1.47 (1.14–1.85)	0.68	n.s.
	MMF	0.45 (0.18–0.82)	**0.49** **(0.37–0.65)**		0.69 (0.57–1.59) ▲ +	**8.07**	**0.018**
Spare respiratory capacity	Control	1.00 (0.68–1.81)	–0.71 (–2.19–0.06) ▲		–1.90 (–2.25–0.57) ▲ +	**16.80**	**<0.001**
	MMF	0.20 (0.00–1.37)	0.03 (–0.27–0.56)		–0.95 (–1.27–0.54) ▲ +	**6.89**	**0.032**

Results are expressed as median and quartiles (in brackets) of OCR-SRC/protein quantification level normalized by vehicle median. AH: aluminum oxyhydroxide (Alhydrogel^®^). V: whole vaccine (EngerixB^®^ 20); *: *p* < 0.05 in Mann–Whitney test between control and MMF groups for treatment indicated in column; ▲: *p* < 0.05 compared to vehicle treatment; +: *p* < 0.05 compared to AH treatment; n.s.: not significant.

**Table A6:** ROS quantification in MMF patients’ and controls’ differentiated PBMCs exposed for 4 h to several treatments.

Treatment		Vehicle	AH	V	H_2_O_2_	AH + H_2_O_2_	V + H_2_O_2_	Friedman	
	Group							Χ²	*p*
ROS	Control	1.00 (0.71–2.35)	1.04 (0.54–1.95)	1.07 (0.47–1.91) ▲	1.99 (1.07–4.47) ▲ + ◆	1.36 (0.67–2.94) + $	1.24 (0.52–2.33) ◆ $	**23.94**	**<0.001**
	MMF	1.04 (0.86–1.29)	0.91 (0.70–1.46)	0.78 (0.61–1.42)	2.04 (0.96–4.58) ▲ + ◆	1.70 (0.61–3.18) $	1.30 (0.64–2.84) $	**16.39**	**0.006**

Results are expressed as median and quartiles (in brackets). Units are standardization of the considered signal by protein quantity normalized by vehicle median. AH: aluminum oxyhydroxide (Alhydrogel^®^); V: whole vaccine (EngerixB^®^ 20); ▲: *p* < 0.05 compared to vehicle treatment; +: *p* < 0.05 compared to AH treatment; ♦: *p* < 0.05 compared to V treatment; $: *p* < 0.05 compared to H_2_O_2_ treatment.
